# Preoperative glycemic response to a very low-calorie diet predicts long-term type 2 diabetes remission after bariatric surgery

**DOI:** 10.1590/0102-67202025000050e1919

**Published:** 2025-12-19

**Authors:** Mariana Fior Frias Thomaz BORTOLAN, Roberto DE CLEVA, Leandro FERREIRA, Denis PAJECKI, Marco Aurelio SANTO

**Affiliations:** 1 Universidade de São Paulo, Faculty of Medicine, Science Program in Gastroenterology – São Paulo (SP), Brazil.; 2 Universidade de São Paulo, Faculty of Medicine, Department of Gastroenterology – São Paulo (SP), Brazil.

**Keywords:** Diabetes Mellitus Type 2, Bariatric Surgery, Gastric Bypass, Diabetes Mellitus Tipo 2, Cirurgia Bariátrica, Obesidade

## Abstract

**Background:**

Obesity and type 2 diabetes mellitus are highly prevalent conditions with a significant public health impact, highlighting the need for effective management strategies. Bariatric surgery is widely recognized for promoting sustained weight loss and high rates of type 2 diabetes mellitus remission.

**Aims:**

This study investigated the preoperative blood glucose response to a very low-calorie diet as a functional predictor of type 2 diabetes mellitus remission following Roux-en-Y gastric bypass.

**Methods:**

198 participants who followed a very low-calorie diet (600 kcal/day) during the preoperative period were included, with glycemic response monitoring.

**Results:**

Complete remission of type 2 diabetes mellitus occurred in 66.7% of patients. Two years after surgery, patients with blood glucose levels below 143 mg/dL on the second day of the very low-calorie diet had a higher likelihood (over 70%) of achieving complete remission type 2 diabetes mellitus in the late postoperative period.

**Conclusions:**

Preoperative capillary blood glucose levels demonstrated good specificity in predicting remissions. These findings reinforce the clinical utility of early glycemic control as a valuable indicator for predicting the success of surgical treatment for type 2 diabetes mellitus.

## ARTICLE HIGHLIGHTS

Obesity and type 2 diabetes mellitus represent major public health challenges, with bariatric surgery recognized as an effective strategy for sustained remission.

This study assessed the predictive value of early glycemic response to a preoperative very low-calorie diet (600 kcal/day) in patients undergoing Roux-en-Y gastric bypass.

Patients achieving capillary blood glucose levels <143 mg/dL on the second day of the diet showed a higher likelihood (>70%) of complete diabetes remission at two years postoperatively.

Preoperative capillary blood glucose demonstrates good specificity and may serve as a practical clinical marker for predicting type 2 diabetes remission following bariatric surgery.

## Summary

**CENTRAL MESSAGE:**

Obesity and type 2 diabetes mellitus (T2DM) are highly prevalent conditions worldwide, with a significant public health impact, reinforcing the need for effective management strategies. Bariatric surgery is widely recognized as an effective treatment for severe obesity and plays a fundamental role in T2DM control.

**PERSPECTIVES:**

There is a significant relationship between the initial glycemic response to dietary intervention and the complete remission of type 2 diabetes mellitus (T2DM) after bariatric surgery. Specifically, capillary blood glucose values equal to or below 143 mg/dL on the second day of a very low-calorie diet serve as a functional predictor of long-term T2DM remission following surgical treatment.

## VISUAL ABSTRACT

**Figure:**
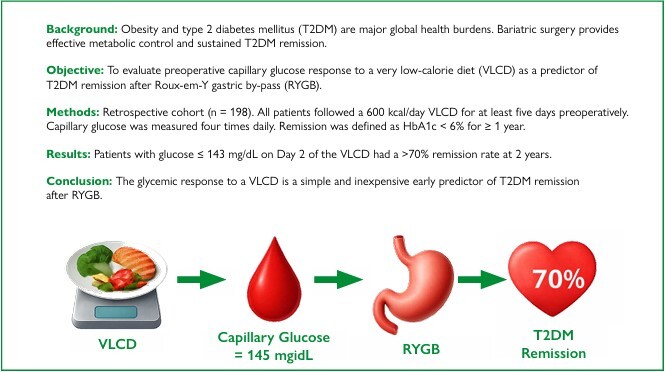

## RESUMO

**Racional:**

A obesidade e o diabetes mellitus tipo 2 são condições altamente prevalentes e com impacto significativo na saúde pública, o que destaca a necessidade de estratégias eficazes para seu manejo. A cirurgia bariátrica é amplamente reconhecida por promover perda de peso sustentada e altas taxas de remissão do diabetes mellitus tipo 2.

**Objetivos:**

Investigar a resposta glicêmica pré-operatória a uma dieta de muito baixa caloria, como preditor de remissão tardia do diabetes mellitus tipo 2, após gastroplastia com derivação em Y-de-Roux.

**Métodos:**

Foram incluídos 198 participantes que seguiram uma dieta de muito baixa caloria (600 kcal/dia) no pré-operatório, com monitoramento da resposta glicêmica.

**Resultados:**

A remissão completa do diabetes mellitus tipo 2 ocorreu em 66,7% dos pacientes. Dois anos após a cirurgia, pacientes com níveis de glicemia abaixo de 143 mg/dl no segundo dia da dieta de muito baixa caloria, tiveram maior probabilidade (acima de 70%) de atingir remissão completa do diabetes mellitus tipo 2 no período pós-operatório tardio.

**Conclusões:**

Os níveis de glicemia capilar pré-operatória demonstraram boa especificidade na predição de remissão. Esses achados reforçam a utilidade clínica do controle glicêmico precoce como indicador viável para prever o sucesso do tratamento cirúrgico do diabetes mellitus tipo 2.

## INTRODUCTION

 Obesity and type 2 diabetes mellitus (T2DM) are highly prevalent conditions worldwide, with a significant public health impact, reinforcing the need for effective management strategies^24,28^. Bariatric surgery is widely recognized as an effective treatment for severe obesity and plays a fundamental role in T2DM control^9,14^. 

 T2DM remission, defined as the reduction or disappearance of disease-related signs and symptoms, is associated with improved physical and mental well-being and has long-term economic and social benefits. The prediction of remission is a relevant factor to consider in the preoperative risk-benefit assessment of bariatric surgery^5,10,23^. 

 Predictive factors for T2DM remission can be categorized into two distinct subgroups. The first relates to the preservation or lesser impairment of pancreatic beta cells, characterized by higher C-peptide levels, shorter diabetes duration, younger age, and adequate glycemic control without the need for insulin. The second subgroup reflects the potential for reducing insulin resistance, represented by a higher preoperative body mass index (BMI), greater visceral fat area, and more significant weight loss after surgery^18^. 

 Several scoring models based on retrospective patient data have been developed to estimate the likelihood of T2DM remission after bariatric surgery, supporting shared decisionmaking between physicians and patients. In Brazil, the most commonly used are the DiaRem, Ad-DiaRem, and ABCD scores. DiaRem considers variables such as age, HbA1c, and use of insulin or other medications, with higher scores indicating lower remission probability. Ad-DiaRem incorporates diabetes duration, enhancing predictive accuracy compared with DiaRem. The ABCD score, based on age, BMI, C-peptide level, and diabetes duration, uses a 0–10 scale in which higher scores predict greater remission probability^6,8,22,23,26^. 

 However, there is still no scientific consensus on the most accurate predictive model for clinical practice. This highlights the importance of ongoing research to refine and validate new tools that can reliably guide patient selection and therapeutic decision-making^6,8,22,25,26^. 

 In this context, the glycemic response to a very low-calorie diet (VLCD) has emerged as an accessible and low-cost clinical indicator. This approach may offer a promising tool to identify individuals more likely to achieve T2DM remission after Roux-en-Y gastric bypass (RYGB). 

 The objective of our study was to evaluate the early effects of a preoperative VLCD on glycemic metabolism as a predictive factor for long-term T2DM remission in patients with severe obesity undergoing RYGB. 

## METHODS

 This retrospective observational cohort study analyzed medical records from 233 patients with T2DM who underwent RYGB at the Bariatric and Metabolic Surgery Unit of Hospital das Clínicas, Universidade de Sao Paulo, from 2017 to 2019. The study was approved by the Ethics Committee of the Institution (Certificate of Presentation for Ethical Appreciation — CAAE: 62964322.7.0000.0068) and did not involve any interventions or interference with patient care. 

 Patients were included if they met the following criteria: BMI>35 kg/m^2^, a preoperative diagnosis of T2DM, aged between 18 and 70 years, adherence to a VLCD for at least five days before surgery, and availability of medical records for at least 12 months postoperatively. Patients with type 1 diabetes or a history of previous bariatric surgery were excluded. 

 All RYGB procedures were performed by a single experienced surgical team at an academic referral center. The procedure involved the creation of a 30 to 40 mL gastric pouch, a 100 cm alimentary limb, and a 100 cm biliopancreatic limb. 

### Data collection

 In the preoperative period, data on clinical history, medication use, biochemical tests, and anthropometric parameters were collected. Patients adhered to a 600 kcal/day VLCD for at least five days before surgery. 

 During hospitalization, glycemic control was assessed through capillary blood glucose measurements taken four times daily, with glycemic management exclusively using regular insulin. 

 In the postoperative period, biochemical parameters — including blood glucose, glycated hemoglobin (HbA1c), and insulin — were measured at six months, one year, and two years after surgery. 

 The criteria for complete remission followed the American Diabetes Association recommendations: fasting glucose <100 mg/dL and HbA1c <6% for one year after RYGB surgery. In addition, the DiaRem score and the ABCD score were calculated for all patients. 

### Statistical analysis

 Continuous variables were assessed for normality using the Anderson-Darling test and for homogeneity of variances using Bartlett’s test. Depending on the data distribution, comparisons were performed using Student’s t-test, Mann-Whitney, or Brunner-Munzel tests, as appropriate. Categorical variables were analyzed using Chi-Square test or Fisher’s Exact test, when applicable. 

 Logistic regression was applied to identify predictors of T2DM remission, defined as HbA1c <6% for at least two years following RYGB. Cut-off points were determined using the Youden method based on the Receiver Operating Characteristic (ROC) curve, and the areas under the curves (AUCs) were compared to assess predictive accuracy. 

## RESULTS

 A total of 198 patients were included who met the inclusion criteria for the study, comprising 153 females (77.3%) and 45 males (22.7%). The mean age of the participants was 52±9.6 years, with a BMI of 44±6.7 kg/m^2^. 

 Preoperative metabolic parameters included a fasting blood glucose level of 170±64.2 mg/dL, a glycated hemoglobin (HbA1c) of 8.5%±2.0, and a C-peptide of 4.3±2.1 ng/mL (Table 1). 

**Table 1:** Clinical and laboratory characteristics of patients in the preoperative period.

Variable	Mean±SD
Age (years)	52±9.6
BMI (kg/m^2^)	44.1±6.7
Glucose (mg/dL)	170±64.2
HbA1c (%)	8.5±2.0
Insulin (mUI/mL)	28.6±17.8
C-Peptide (ng/mL)	4.3±2.1
Cholesterol (mg/dL)	179.2±45.7
HDL (mg/dL)	41.9±9.6
LDL (mg/dL)	108.6±40.7
VLDL (mg/dL)	28.5±10.2
Triglycerides (mg/dL)	162.7±75.2

SD: standard deviation; BMI: body mass index; HbA1c: glycated hemoglobin; HDL: high-density lipoprotein cholesterol; LDL: low-density lipoprotein cholesterol; VLDL: very-low-density lipoprotein cholesterol.

 Most patients (70.1%) had been diagnosed with T2DM for five or more years, and 71% had a C-peptide level=3 ng/mL. Most patients (68.1%) were on multiple oral hypoglycemic agents, while a smaller proportion (33.8%) required insulin therapy. 

**Figure 1:**
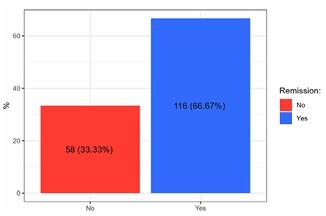
Complete type 2 diabetes mellitus remission in the second postoperative year.

 Of the 198 medical records analyzed, 174 had biochemical data on glycated hemoglobin (HbA1c) in the second postoperative year. Among these, 116 patients (66.7%) achieved complete T2DM remission, defined as HbA1c<6% (Figure 1). There was a reduction in the mean BMI of patients (44.1±6.7 to 31.5±4.1 kg/m2) and the mean HbA1c (8.5±2.1 to 5.8±0.8%). 

**Figure 2:**
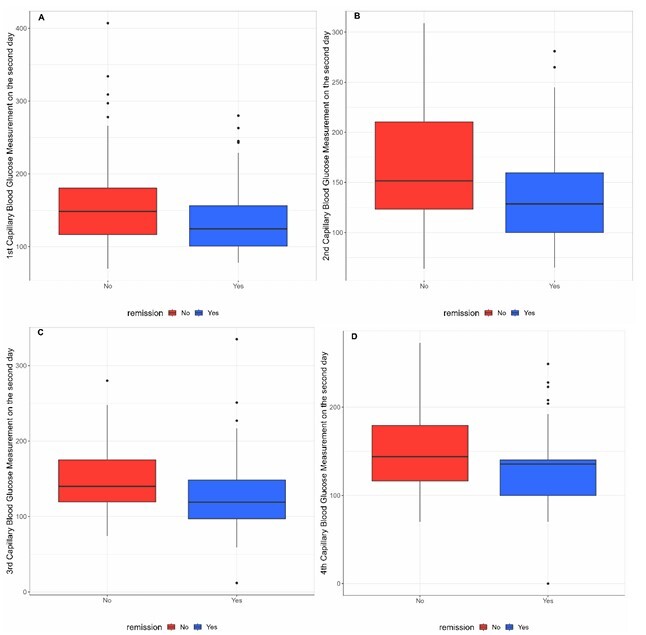
Capillary blood glucose levels on the second preoperative day during a very low-calorie diet in the complete remission and non-remission groups. A) First capillary blood glucose measurement on the second day of a very low-calorie diet in the complete remission and non-remission groups; B) Second capillary blood glucose measurement on the second day of a very low-calorie diet in the complete remission and non-remission groups; C) Third capillary blood glucose measurement on the second day of a very low-calorie diet in the complete remission and non-remission groups; D) Fourth capillary blood glucose measurement on the second day of a very low-calorie diet in the complete remission and non-remission groups).

**Figure 3:**
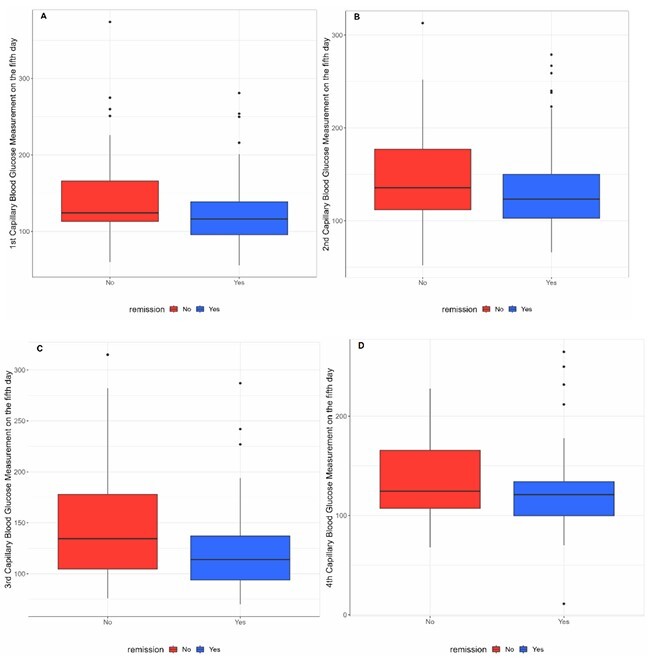
Capillary blood glucose levels on the fifth preoperative day during a very low-calorie diet in the complete remission and non-remission groups. A) First capillary blood glucose measurement on the fifth day of a very low-calorie diet in the complete remission and non-remission groups; B) Second capillary blood glucose measurement on the fifth day of a very low-calorie diet in the complete remission and non-remission groups; C) Third capillary blood glucose measurement on the fifth day of a very low-calorie diet in the complete remission and non-remission groups; D) Fourth capillary blood glucose measurement on the fifth day of a very low-calorie diet in the complete remission and non-remission groups.

 Preoperative capillary blood glucose levels during the VLCD were consistently lower in the group that achieved complete T2DM remission, as illustrated in Figures 2 and 3. 

 Cut-off points were determined using the Youden method based on the ROC curve to evaluate the predictive capacity of the glycemic response to a VLDC in relation to T2DM remission two years after RYGB. The glycemic response on the second and fifth days of VLCD was compared with the ABCD scores (age, BMI, C-peptide level, and duration of diabetes) and DiaRem score (glycated hemoglobin, age, use of insulin and use of oral hypoglycemic agents)^6,10,26^. 

 There were significant contributions to the capillary blood glucose (Dextro) response to the very low-calorie diet, as well as to the ABCD and DiaRem scores, as presented in Table 2. 

**Table 2:** Predictive capacity of ABCD, DiaRem, and capillary blood glucose levels following a very low-calorie diet.

Variable	Category	No (%)	Yes (%)	p-value
ABCD	=5	43 (44.3)	54 (55.7)	<0.001
ABCD	>5	8 (13.6)	51 (86.4)	
DiaRem	=13	17 (19.3)	71(80.7)	<0.001
DiaRem	>13	29 (63)	17 (37)	
Dextro Day 2	=143	28 (26.2)	79 (73.8)	0.013
First Measure	>143	30 (44.8)	37 (55.2)	
Dextro Day 2	=143	25 (25.5)	73 (74.5)	0.015
Second Measure	>143	33 (43.4)	43 (56.6)	
Dextro Day 2	=143	32 (27.6)	84 (72.4)	0.027
Third Measure	> 143	26 (44.9)	32 (55.1)	
Dextro Day 2	=143	29 (24.6)	89 (75.4)	0.001
Fourth Measure	>143	29 (51.8)	27 (48.2)	
Dextro Day 5	=143	37 (29.1)	90 (70.9)	0.07
First Measure	>143	22 (44.7)	26 (55.3)	
Dextro Day 5	=143	32 (28.1)	82 (71.9)	0.06
Second Measure	>143	27 (43.3)	34 (56.7)	
Dextro Day 5	=143	32 (25.6)	93 (74.4)	0.001
Third Measure	>143	26 (53.1)	23 (46.9)	
Dextro Day 5	=143	34 (26.6)	94 (73.4)	0.003
Fourth Measure	>143	24 (52.2)	22 (47.8)	

ABCD score: age; BMI: C-peptide level, and duration of diabetes; DiaRem score: glycated hemoglobin, age, use of insulin and use of oral hypoglycemic agents; Dextro: capillary blood glucose.

**Figure 4:**
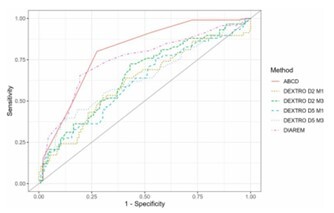
Receiver Operating Characteristic Curve for DiaRem, ABCD, and capillary blood glucose following a very low-calorie diet.

 The area under the curve (AUC) of the ROC curve was used to evaluate the specificity of capillary blood glucose measurements, as well as the ABCD and DiaRem scoring systems. Although the ABCD and DiaRem scores exhibited higher AUC values, comparisons of these curves demonstrated that the newly proposed predictor also had good specificity (Table 3 and Figure 4). 

**Table 3:** Area Under the Curve (AUC) for DiaRem, ABCD, and Capillary Blood Glucose.

Criteria	AUC	Sensitivity	Specificity
DiaRem=13	0.75	0.66	0.84
ABCD>5	0.8	0.72	0.88
Dextro<143 Day 2 – First Measure	0.62	0.53	0.71
Dextro<143 Day 2 – Second Measure	0.66	0.57	0.74
Dextro<143 Day 2 – Third Measure	0.66	0.57	0.74
Dextro<143 Day 2 – Fourth Measure	0.64	0.55	0.73
Dextro<143 Day 5 – First Measure	0.62	0.53	0.71
Dextro<143 Day 5 – Second Measure	0.6	0.51	0.69
Dextro<143 Day 5 – Third Measure	0.65	0.55	0.74
Dextro<143 Day 5 – Fourth Measure	0.6	0.5	0.69

ABCD score: age, BMI, C-peptide level, and duration of diabetes; DiaRem score: glycated hemoglobin, age, use of insulin and use of oral hypoglycemic agents); Dextro: capillary blood glucose.

## DISCUSSION

 Obesity is one of the main causes of the global increase in the prevalence of T2DM, making weight loss strategies essential for disease control^2,19,21^. Bariatric surgery, particularly using techniques such as RYGB and sleeve gastrectomy (SG), has proven to be highly effective in metabolic control and T2DM remission^1,3,19^. However, the response to surgery varies among individuals, reinforcing the need for predictive tools such as the DiaRem and ABCD scores to identify patients with a higher likelihood of remission^7,11^. 

 This study demonstrated that preoperative capillary blood glucose levels after a VLCD can serve as a predictor of T2DM remission. The findings allowed accurate prediction of remission in over 70% of cases, emphasizing the clinical utility of this approach, despite its limitations. 

 VLDC are also effective in improving glycemic control and restoring pancreatic function, making them beneficial in both surgical and non-surgical contexts^4,16,17,20^. Glycemic control achieved through a VLCD is associated with a drastic reduction in caloric intake, leading to a rapid decrease in lipotoxicity and an increase in insulin sensitivity. This metabolic improvement occurs due to the reduction of hepatic and pancreatic fat stores, resulting in better insulin secretion by pancreatic beta cells^12,13,18^. 

 In contrast, the long-term glycemic control promoted by bariatric surgery involves additional mechanisms, including hormonal changes that regulate glucose metabolism. After surgery, incretin hormone levels, such as glucagon-like peptide-1 (GLP-1) and peptide YY (PYY), increase, enhancing insulin secretion and improving postprandial glycemic response^1,15,19^. 

 However, while caloric restriction effectively promotes weight loss and glycemic remission, maintaining these effects long term presents significant challenges. Biological factors, such as a history of higher body weight throughout life, and psychological factors, including dichotomous thinking, are associated with weight regain^7^. Additionally, hormonal adaptations to caloric restriction, such as reduced amylin and GLP-1 levels and increased ghrelin secretion, further contribute to weight regain^15,25,27^. 

 An increasing number of studies have shown that factors such as shorter T2DM duration, better preoperative glycemic control, lower baseline glycated hemoglobin, reduced waist circumference, and greater postoperative weight loss are associated with higher remission rates and a lower risk of recurrence^25,27^. In our study, these patterns were confirmed, with statistically significant differences observed in preoperative capillary blood glucose, fasting blood glucose, glycated hemoglobin, and C-peptide levels between patients who achieved complete remission and those who did not two years postoperatively. These results reinforce that better preoperative glycemic control indicators are associated with an increased likelihood of T2DM remission following RYGB. 

## CONCLUSIONS

 There is a significant relationship between the initial glycemic response to dietary intervention and the complete remission of T2DM after bariatric surgery. Specifically, capillary blood glucose values equal to or below 143 mg/dL on the second day of a very low-calorie diet serve as a functional predictor of long-term T2DM remission following surgical treatment. 

## Data Availability

The datasets generated and/or analyzed during the current study are available from the corresponding author upon reasonable request.
